# A genetic profile of hereditary neuropathies in two Southern African cohorts

**DOI:** 10.3389/fneur.2026.1918540

**Published:** 2026-10-07

**Authors:** Tarin Europa, Stanley Zimba, Niki Floudiotis, Somwe Wa Somwe, David Bearden, Musambo Kapapa, Rita Horvath, Lindsay Wilson, Natalia Dominik, Michael Hanna, Mary M. Reilly, Christopher J. Record, Katherine Schon, Saifeldeen M. Elshahawy, Nabeelah Peerbhai, Jeannine M. Heckmann, Michelle Kvalsund, Melissa Nel

**Affiliations:** 1 UCT Neuroscience Institute, University of Cape Town, Cape Town, South Africa; 2 Neurology Research Group, Department of Medicine, University of Cape Town, Cape Town, South Africa; 3 Department of Internal Medicine, University of Zambia School of Medicine, Lusaka, Zambia; 4 Department of Paediatrics and Child Health, School of Medicine and Health Sciences, University of Lusaka, Lusaka, Zambia; 5 Department of Neurology, University of Rochester Medical Center, Rochester, NY, United States; 6 Department of Physiotherapy, University of Zambia School of Health Sciences, Lusaka, Zambia; 7 Department of Clinical Neurosciences, University of Cambridge, Cambridge, United Kingdom; 8 Department of Neuromuscular Diseases, UCL Queen Square Institute of Neurology, University College London, London, United Kingdom; 9 Department of Genomic Medicine, University of Cambridge, Cambridge, United Kingdom; 10 Clinical Omics and Informatics (COIN) Unit, UCT Neuroscience Institute, University of Cape Town, Cape Town, South Africa; 11 African Bioinformatics Institute, Cape Town, South Africa

**Keywords:** Africa, Charcot–Marie–Tooth (CMT), genetic profile, hereditary neuropathy, mutational profile

## Abstract

**Introduction:**

Data on hereditary neuropathies in African populations are sparse. In sub-Saharan Africa, genetic testing for Charcot–Marie–Tooth (CMT)-related neuropathies has been largely limited to *PMP22* screening for demyelinating CMT.

**Aim:**

We report the genetic profiles of individuals with hereditary neuropathies enrolled at two centers in Cape Town, South Africa, and Lusaka, Zambia.

**Methods:**

Between 2019 and 2024, consecutive patients with suspected CMT were enrolled in the International Centre for Genomic Medicine in Neuromuscular Diseases (ICGNMD) consortium. The Cape Town center also enrolled patients locally between 2017 and 2019. Electrophysiological testing categorized participants into demyelinating CMT (CMT1; median motor nerve conduction velocity (CV) ≤ 25 m/s), axonal CMT (CMT2; >45 m/s), or intermediate CMT (CMTi; >25; ≤45 m/s). *PMP22* assays were performed in probands with CMT1/CMTi, while *RFC1* assays were performed in adults with sensory ataxic neuropathy (SAN). All other participants underwent whole exome sequencing (WES) or whole genome sequencing (WGS).

**Results:**

The 68 probands had a median age at symptom onset of 13 years (interquartile range 6;26). In total, 32 (47%) had CMT2, seven (10%) had CMT1, seven (10%) had CMTi, and the remaining (32%) had sensory, sensory ataxic, motor, or complex neuropathies. Genetic diagnoses were determined in 54% of probands. All five individuals with CMT1 and *PMP22* duplications had European ancestry, except for one individual with admixed ancestry. CMT2 was most frequently caused by heterozygous pathogenic or likely pathogenic variants in *MFN2* (22%). Compound heterozygous *MPV17* variants (p.Gln36Ter; p.Arg125Trp) were found in four probands of African ancestry with CMT2 or CMTi. Single cases categorized as CMT2 had single variants in 13 different genes. Furthermore, one individual with late-onset CMTi had an *MPZ* variant. In total, four “complex” neuropathies had variants in *PEX11B, SERAC1, TDP1,* and *ATM*. Homozygous *TDP1* p.His493Arg variants were shared between two unrelated probands of African ancestry with complex sensory ataxic neuropathy. One family of European ancestry had pseudodominant homozygous *RFC1* expansions.

**Conclusion:**

The genetic profile of CMT in Southern Africa differs from that reported elsewhere. *MFN2-*assocaited CMT2 was the most common subtype. CMT1 was absent in individuals of African ancestry. *MPV17*-associated neuropathy was found in CMT2/CMTi cases from South Africa.

## Highlights

- The most common CMT subtypes in Southern Africa differ from those reported globally.
- Demyelinating neuropathies (CMT1) were rare among patients of African ancestry with hereditary neuropathies.
- CMT2 related to *MFN2* variants was the most common CMT subtype.
- MPV-associated neuropathy was a frequent cause of CMT/CMTi among South African individuals.

## 1 Introduction

Charcot–Marie–Tooth (CMT) disease and related hereditary neuropathies comprise a variety of clinical phenotypes, categorized by their varying degree of motor and/or sensory involvement and/or association with other neurological or multisystem involvement (complex neuropathy syndromes) (1). Sensorimotor neuropathy is the most common form of CMT and can be further categorized based on nerve conduction velocity (CV) into demyelinating CMT (CMT1), axonal CMT (CMT2), and intermediate CMT (CMTi). In cohorts from the United Kingdom (UK) and North America, demyelinating CMT1 constitutes between 41 and 64% of cases, while CMTi accounts for 14% and axonal forms (CMT2) for 19–27% (2, 3). The frequencies from two cohorts in Brazil, originating from the Southern- and Central-West regions, respectively, ranged between 70 and 80% for CMT1/CMTi (demyelinating and intermediate together) and between 20 and 22% for CMT2 (4). Therefore, the distribution of CMT in the Northern Hemisphere and parts of South America appears similar, with CMT2 representing up to one-quarter of CMT cases. In China and Taiwan, 47–73% of cases were classified as CMT1/CMTi, while 26–30% were classified as CMT2 (5, 6).

Furthermore, *PMP22* duplications are the predominant molecular cause of CMT, accounting for 35–57% of diagnoses in cohorts comprising participants from the UK and China (3, 5). This is not unexpected since CMT1 is the predominant phenotype and 67–81% of CMT1 cases of European ancestry are due to *PMP22* copy number variants (CNVs) (1–3, 7). Among CMT1 cases of Chinese ancestry, *PMP22* CNVs account for 35–66% (5, 6).

Very little is known about CMT in Africa (8, 9). In North Africa, the most common CMT presentations were demyelinating autosomal recessive neuropathies (9). In a previous, smaller South African adult cohort of 31 probands with hereditary neuropathies, no demyelinating neuropathies or *PMP22* CNVs were reported among patients of African genetic ancestry (10). Overall, *MFN2* mutations accounted for 10% of CMT cases in patients of African genetic ancestry (10). A report of a mixed-ancestry cohort of 33 children with CMT from South Africa who underwent genetic testing found two cases of African ancestry with *PMP22* CNVs and reported similar frequencies across self-reported ancestry groups (11). Nevertheless, these findings suggest that the Southern African genetic profile of CMT may differ from that reported elsewhere in the world.

One of the reasons for the sparse data from Africa is limited access to genetic testing. Diagnostic genetic testing for hereditary neuropathies in sub-Saharan Africa has been largely limited to a multiplex ligation-dependent probe amplification (MLPA) assay used to detect CNVs in *PMP22.* Isolated diagnostic laboratories offered this testing, primarily through the South African National Health Laboratory Service (NHLS), but it required out-of-pocket payment. Although CNVs in *MPZ* and *GJB1* were also included, these are estimated to account for <2% of pathogenic variants in European CMT cohorts (7).

We aimed to describe the mutational profile of two Southern African cohorts of patients with clinical phenotypes suggestive of CMT. The International Centre for Genomic Medicine in Neuromuscular Diseases (ICGNMD) offered next-generation sequencing and other assays, as indicated, for cases without a genetic diagnosis (12). Here, we describe the clinical and genetic profiles of these cohorts, whose participants underwent research-based genetic testing after locally available MLPA *PMP22* assays were negative, when appropriate.

## 2 Patients and methods

### 2.1 Patient selection

Patients suspected of having CMT were enrolled from 2017 to 2019 in a local study at Groote Schuur Hospital (GSH) in Cape Town, South Africa. Between 2019 and 2024, patients from GSH in Cape Town, South Africa, and the University Teaching Hospital in Lusaka, Zambia, were enrolled as part of the ICGNMD study. The studies were approved by the Human Research and Ethics Committee [the University of Cape Town (UCT) and the University of Zambia (UNZA)] (UCT HREC R030/2018 and UNZA BREC 091-2019). All patients provided consent for the recording and sharing of anonymized clinical and genetic data. The inclusion criteria were a chronic, progressive history of peripheral sensory and/or motor dysfunction with or without foot deformities (including pes cavus), impaired deep tendon reflexes, distal weakness, distal atrophy, and length-dependent loss of sensation. Electrophysiological studies were performed to differentiate between demyelinating and axonal neuropathies and were categorized as described in a previous study (3). All patients were required to self-report their ancestry as African, European, Asian, Indian, or admixed. In South Africa, these subpopulation categories represent the national census categories. A proportion of the GSH cohort (*n* = 16) was previously described (10).

### 2.2 Clinical data collection

Participants underwent detailed neurological examinations (JMH/NF/MK/SZ), and de-identified phenotypic data were recorded systematically using a standardized template. Participants were categorized into CMT subtypes [Table 1; adapted from Record et al. (3)].

**Table 1:** Categorization of Charcot–Marie–Tooth (CMT) disease by clinical and electrophysiological criteria.

CMT phenotype	Electrophysiological criteria
CMT1	Demyelinating neuropathy with upper limb MNCV ≤25 m/s
CMT2	Axonal neuropathy with upper limb MNCV >45 m/s
CMTi	Intermediate conduction slowing with upper limb MNCV >25 but ≤ 45 m/s
HSN	Sensory neuropathy (clinically and/or impaired peak sural sensory nerve action potentials); presenting features such as ulceration and painless fractures; absent motor involvement on electrophysiological assessment
HMN	Motor neuropathy clinically and with impaired motor conduction; normal sensory nerve action potentials on electrophysiological assessment
SAN	Isolated sensory ataxic neuropathy (SAN) or SAN with non-neuropathic features (cerebellar/vestibular)
“complex”	Complex neuropathy (sensorimotor, sensory, or motor) with demyelinating, intermediate, or axonal distal neuropathic features on electrophysiological assessment, together with at least one non-neuropathic manifestation* (significant upper motor neuron involvement, intellectual disability, and cerebellar ataxia).
HNPP	Clinical phenotype of recurrent mononeuropathies at compression sites without an alternate acquired cause, with or without underlying electrophysiological abnormalities

Participants were categorized according to clinical and electrophysiological neuropathy features. CMT refers to clinical sensory and motor impairment in addition to electrophysiological features. Motor nerve conduction velocity (MNCV) is reported in meters per second (m/s). Upper limb MNCV refers to the median nerve results. Intermediate (i). Hereditary sensory neuropathy (HSN). Hereditary motor neuropathy (HMN). Sensory ataxic neuropathy (SAN). Hereditary neuropathy with liability to pressure palsy (HNPP). *All but one participant had at least two non-neuropathic manifestations. Adapted from (3).

### 2.3 Genetic testing

Participants with motor nerve conduction velocities in the demyelinating or intermediate range first underwent *PMP22* copy number variant testing (*n* = 11) (SALSA MLPA P405 CMT1, MRC Holland, Amsterdam), with additional CNV probes for *GJB1* and *MPZ* performed at the South African NHLS. For the remaining phenotypes and *PMP22* CNV-negative cases, DNA extracted from whole blood was sent for whole exome sequencing (WES) or *RFC1* testing, as clinically appropriate (performed at University College London, ND).

*RFC1* repeat-primed PCR (RP-PCR) testing was performed as previously described (13). Briefly, genomic DNA was subjected to flanking PCR and RP-PCR for the canonical AAGGG, AAAGG, and AAAAG configurations. Provided that enough high-quality DNA was available, samples were analyzed by Southern blotting, as previously described (13), to confirm the presence of biallelic expansions.

### 2.4 Next-generation sequencing

WES libraries were generated using the SureSelect All Exon V6 (Agilent) kit, and samples were sequenced on the Illumina NovaSeq 6,000 instrument to a minimum coverage of 100X and a read length of 150 bp, as previously described (12). Whole genome sequencing (WGS) was undertaken opportunistically in a subset of isolated cases, depending on local test availability (10) or via the ICGNMD, rather than as part of a tiered testing strategy. For whole genome sequencing (WGS), libraries were generated using the MGIEasy Universal DNA Library Prep Kit, and samples were sequenced on the Illumina NovaSeq 6000 instrument to a minimum coverage of 30× and a read length of 150 bp. PacBio HiFi long-read WGS was performed in one case according to the methods described previously (14).

### 2.5 Bioinformatics analysis

FASTQ files were aligned to the GRCh38 reference genome using either a linear or personal pangenome reference workflow (15) on Ilifu, a regional high-performance computing infrastructure in Cape Town, South Africa.^1^ Single-nucleotide variants (SNVs) and small insertions/deletions (indels) were called using DeepVariant (v.1.6.0). Following variant calling, variant call format (VCF) files were uploaded to Franklin by Genoox^2^ for AI-assisted variant prioritization using Human Phenotype Ontology (HPO) terms relevant to each case. AI-based prioritization was used as a guide rather than a primary filter. In every case, two independent analysts manually reviewed variants in clinically relevant gene panels appropriate to the patient’s phenotype to ensure that diagnostically important variants were not overlooked. The analysis focused on short variants (SNVs and small indels); copy number variants and variants in the mitochondrial genome were not assessed. Variants were not confirmed by Sanger sequencing. Instead, calls were quality controlled using read depth, mapping quality, and allele fraction, and only those meeting pre-specified thresholds (read depth ≥ 20X, MAPQ≥ 20, allele fraction 0.35–0.65 for heterozygous and ≥ 0.90 for homozygous) were retained.

### 2.6 Variant curation, classification, and ClinVar submission

Variants identified through genomic sequencing were curated and classified using the ClinGen Variant Curation Interface (16). Variants were interpreted according to the American College of Medical Genetics and Genomics/Association for Molecular Pathology (ACMG/AMP) v3 guidelines (17), incorporating recommendations from the Clinical Genome Resource Sequence Variant Interpretation Working Group, as well as gene- and variant-level specifications from ClinGen Expert Panels where available. Population frequency data were assessed using gnomAD v4, the African Genome Variation Database, and an internal African control dataset to inform rarity assessments. Existing ClinVar submissions and the published literature were reviewed as part of the curation process. *In silico* prediction tools were used as supporting evidence where applicable. Variants were classified as likely pathogenic, pathogenic, and variants of uncertain significance (VUSs) according to ACMG criteria. Where a VUS showed strong phenotypic concordance and biological plausibility, it was retained as a candidate explanatory variant and reported as such, while retaining its formal classification as a VUS. These variants were also submitted to ClinVar, with the inclusion of variant- and case-level information as previously described (18). For certain cases, especially where there were difficulties in interpreting the pathogenicity of variants, a virtual multidisciplinary meeting involving clinicians, geneticists, and bioinformaticians from African and UK sites was conducted.

### 2.7 Statistical analysis

De-identified clinical data were exported from REDCap to Microsoft Excel. Age at onset and other continuous variables with non-normal distributions were reported as medians and interquartile ranges and compared using the Mann–Whitney *U* test (Prism Graphpad v10). Categorical variables were reported as percentages and compared using the chi-squared test or Fisher’s exact test, as appropriate. *p*-values of <0.05 were considered significant.

## 3 Results

The cohort comprised 68 probands: 52 probands from South Africa (SA) and 16 probands from Zambia (ZM). The median age at onset of neuropathy symptoms was 13 years (interquartile range (IQR), 6; 26), with a significantly younger age at onset for ZM cases [3.5 years (IQR 2; 9.8)] compared to SA cases [16 years (IQR 10; 32)] (*p* < 0.0001) (Table 2). A family history of hereditary neuropathy was present in 50% of SA cases and 43% of ZM cases (*p* = 0.66). Consanguinity was only reported in two SA families.

**Table 2:** Demographics and neuropathy categories of South African and Zambian probands.

Demographics	Total (*n* = 68)	SA (*n* = 52)	ZM (*n* = 16)	*p*-value
Male sex (%)	40	29 (56)	11 (69)	0.36
Median age of onset (IQR)	13 (6; 26)	16 (10;32)	3.5 (2;10)	**<0.0001**
Positive family history (%)	33	26 (50)	7 (43)	0.66
Solved cases (%)	37 (54)	28 (54)	9 (56)	0.87
CMT phenotype (*n*, %)				
CMT1	7 (10)	7 (13)	0	—
CMT2	32 (47)	21 (40)	11 (69)	**0.047**
CMTi	7 (10)	6 (12)	1 (6)	>0.99
HMN	6 (9)	3 (6)	3 (19)	0.14
HSN	3 (4)	3 (6)	0	—
SAN	4 (6)	4 (8)	0	—
HNPP	0	0	0	—
Complex	9 (13)	8 (17)	1 (6)	0.67

Numbers in brackets indicate the percentage (%) of the column total. South Africa (SA), Zambia (ZM). Interquartile range (IQR); Charcot–Marie–Tooth (CMT); hereditary sensory neuropathy (HSN); hereditary motor neuropathy (HMN); sensory ataxic neuropathy (SAN); hereditary neuropathy with liability to pressure palsy (HNPP); “complex” refers to cases with at least one additional non-neuropathic symptom. Significant *p*-values are indicated in bold text (*p* < 0.05).

Participants from both cohorts were categorized into CMT and other neuropathy phenotypes as described in Table 1. The phenotypic distribution was as follows: CMT2, 47%; CMT1, 10%; CMTi, 10%; hereditary motor neuropathy (HMN), 9%; hereditary sensory neuropathy (HSN), 4%; sensory ataxic neuropathy (SAN), 6%; and complex neuropathies, 13%. A genetic diagnosis was determined in 54% of participants overall (Supplementary Table 1). Self-reported ancestry by neuropathy phenotype, together with pathogenic variation with ClinVar accession numbers where applicable, is denoted in Supplementary Table 1.

### 3.1 Sensorimotor neuropathies (CMT1, CMT2, and CMTi)

Demyelinating sensorimotor neuropathies, with conduction velocities (CVs) ≤ 25 m/s as described in Record et al. (3), were uncommon and occurred only among seven SA participants who self-reported as European (*n* = 6/7) or of admixed European ancestry (*n* = 1/7). Therefore, CMT1 accounted for 10% of the overall cohort. Of these CMT1 probands, five (71%) were found to have 1.5 Mb *PMP22* duplications (Figure 1A), while two remained without a genetic diagnosis despite short-read WGS.

**Figure 1:**
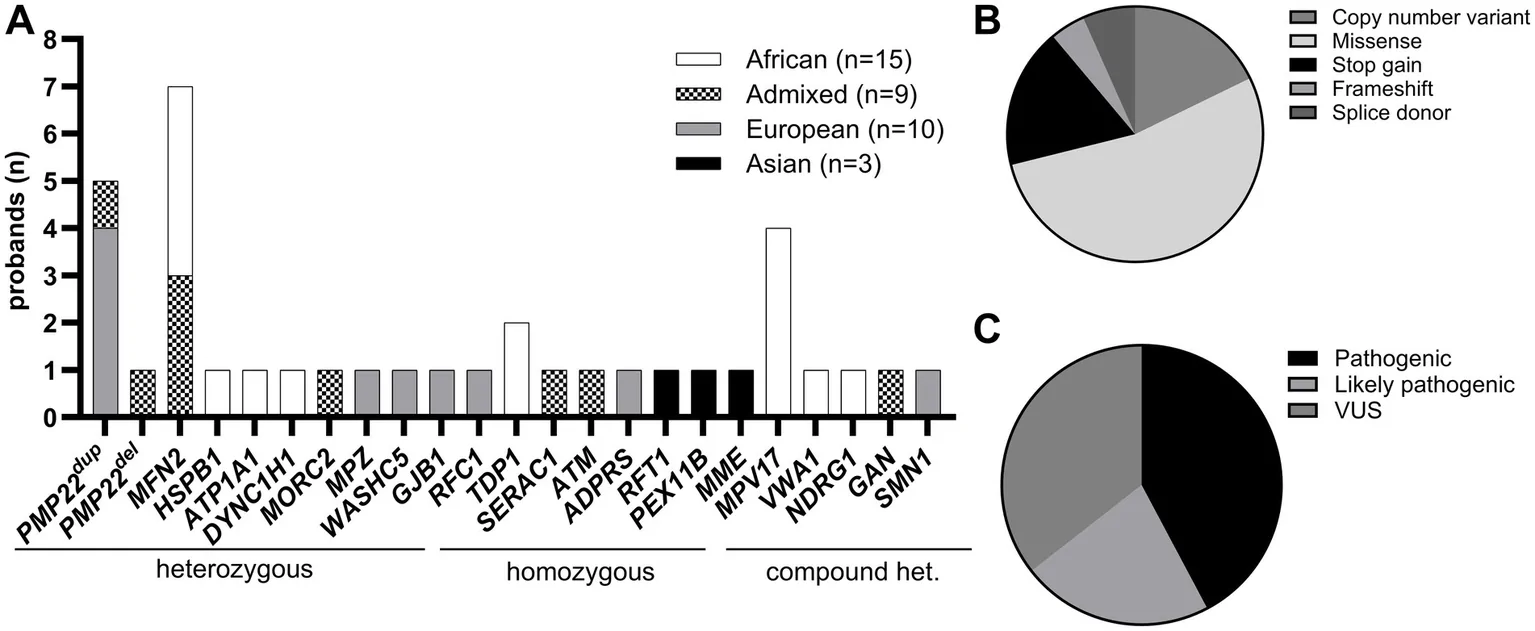
Mutational profile of genetic neuropathies across the two Southern African cohorts. **(A)** Probands with genetic diagnoses (*n* = 37) categorized by genomic inheritance and self-reported ancestry; African (AFR), South African admixed (ADM), European (EUR), or Asian (ASI, including South Asian) ancestry. Heterozygous (het.). **(B)** Variant types (mechanism). **(C)** American College of Medical Genetics and Genomics (ACMG) classification. dup refers to duplication, del refers to deletion.

In contrast, CMT2 was the most frequent category of CMT, representing 47% of the overall sample and the most common category in both SA and ZM cohorts independently (40 and 69% from SA and ZM sites, respectively). *MFN2* gene variants were the most frequent in this group (7/32, 22%; Figure 1A). The pathogenic variants identified were NM_014874.4(*MFN2*):c.281G > A (p.Arg94Gln), c.775C > T (p.Arg259Cys), c.839G > A (p.Arg280His), c.1090C > T (p.Arg364Trp), and c.280C > T (p.Arg94Trp). The likely pathogenic variants were c.616A > G (p.Thr206Ala) and c.1083C > G (p.His361Gln). Hyperreflexia and spasticity were observed in two SA probands, while another developed adult-onset blindness due to optic atrophy. No upper motor neuron signs or optic atrophy were noted in the ZM probands. In total, four individual cases of autosomal dominant axonal neuropathies were identified (*PMP22* deletion, *ATP1A1*, *HSPB1,* and *DYNC1H1*). The sporadic SA proband of admixed European ancestry with a *PMP22* deletion was categorized as CMT2 according to the electrophysiological criteria in Table 1. She had no history of hereditary neuropathy with liability to pressure palsy (HNPP), although pain was a prominent clinical feature. The remaining molecular diagnoses in the CMT2 group comprised two probands with compound heterozygous *MPV17* variants that, despite a VUS classification, were nevertheless strong phenotype-matched candidates for autosomal recessive *MPV17*-associated neuropathy (Figure 1A; Supplementary Tables 2–4). Autosomal recessive neuropathies were also found in *GAN*, *RFT1,* and *ADPRS* (Figure 1A). In *GAN*, the compound heterozygous variants NM_022041.4(*GAN*):c.280C > T (p.Gln94Ter) and c.944C > T (p.Pro315Leu) were classified as VUSs (Supplementary Table 4). Given the concordant phenotype and the established gene–disease association, we considered this the most likely explanation for the participant’s presentation. A single proband was found with compound heterozygous *SMN1* variants, comprising an SMN1 deletion in *trans* with a missense variant (pathogenic SMN1 p.Ile116Thr, Supplementary Table 1). She was clinically categorized as CMT2 as the best fit according to the criteria in Table 1, based on electromyographic evidence of an axonopathy and absent sural sensory nerve action potentials (SNAPs), despite a normal sensory examination. Using long-read WGS, the pathogenic p.Ile116Thr was uncovered in the participant’s single copy of *SMN1* [reported in (14)]. The genetic diagnosis was therefore revised to SMA type 3. For consistency, we retained the *a priori* phenotypic classification for this participant, as shown in Table 1.

In a sporadic participant of African ancestry with childhood-onset severe neuropathy, compound heterozygous VUSs were identified in *NDRG1*, consistent with autosomal recessive CMT4D (see Supplementary Table 1). However, Sanger sequencing confirmation of the *trans* configuration was not performed because paternal DNA was not available. Clinically, this proband did not have hearing loss at the time of the last follow-up, at the age of 19. Electrophysiological classification was uncertain, as no responses were elicited in either the upper or lower limbs on nerve conduction studies (absent sensory and motor nerve action potentials), but electromyography showed chronic active denervation, which was more likely to be primary axonal. Although we acknowledge that the electrophysiological classification was not definitive and could have been secondary to severe demyelination, severe childhood-onset CMT2 cases frequently had unobtainable motor responses in the upper extremities (19).

Interestingly, in addition to the two CMT2 probands with candidate compound heterozygous *MPV17*-associated neuropathy VUSs—NM_002437.5(*MPV17*):c.106C > T (p.Gln36Ter) and c.373C > T (p.Arg125Trp)—a further two probands with intermediate-range conduction velocities (median nerve conduction velocities ranging from 34.5to 43.7 m/s) also harbored p.Gln36Ter and p.Arg125Trp (Figure 1A; Supplementary Tables 2–4). These were confirmed to be in *trans* in one of the four probands by long-read WGS. All four individuals with likely *MPV17*-associated neuropathy had juvenile symptom onset of walking difficulties with foot drop, later followed by distal hand weakness (Supplementary Table 2). All were areflexic and had distal amyotrophy. Furthermore, two individuals had numbness in their hands and feet, although sural sensory nerve action potentials were absent in all four individuals (electrophysiological data are summarized in Supplementary Table 3). Only one proband had pes cavus, clawing of the toes, and sensorineural deafness. None of the individuals had evidence of chronic liver disease or abnormal liver enzyme levels. None of these individuals were known to be related, and none of their families had consanguineous marriages.

The remaining probands with CMTi included one with late-onset autosomal dominant neuropathy and a VUS in *MPZ,* NM_000530.8(MPZ):c.212A > G (p.Glu71Gly), and another with no family history of neuropathy and a pathogenic *MORC2* variant NM_001303256.3(MORC2):c.754C > T (p.Arg252Trp). Of interest, X-linked *GJB1* neuropathy was found in a proband of European ancestry (Figure 1A). One ZM proband of self-reported Asian ancestry had late-onset CMT (onset at 49 years) and a homozygous *MME* variant (NM_007289.4(MME):c.2170C > T(p.Gln724Ter)), which was considered likely pathogenic. The proband had an affected sibling with the same homozygous variant. Consanguinity was not reported.

Although we used a recent, more stringent phenotypic classification of demyelinating neuropathy (CV ≤ 25 m/s) and intermediate neuropathy (CMTi) (CV > 25 m/s but ≤45 m/s) (3), older classifications (19) defined demyelinating neuropathy as CV ≤ 35 m/s and intermediate neuropathy as CV > 35 m/s but ≤45 m/s. The seven participants classified herein as having CMTi (Supplementary Table 1), with CVs > 25 m/s but ≤45 m/s, had median motor nerve CVs ranging between 34.5 and 43.7 m/s and an average CV of 37.4 m/s (SD ± 4.1). However, if a less stringent definition of slow CVs were applied in this cohort, such that all CVs ≤ 35 m/s were considered demyelinating [as in Saporta et al. (19)], this cohort would include an additional three participants classified as having CMT1, bringing the total to 14.7% (10/68), and *PMP22* CNVs would account for 60% (6/10). The three additional participants with median nerve CVs ≤ 35 m/s were all considered to have a genetic diagnosis, involving *MME* (median motor nerve CV = 34 m/s), *MPV17* (CV = 34.5 m/s), and *MORC2* (CV = 34.7 m/s).

### 3.2 Hereditary motor neuropathy (HMN) and hereditary sensory neuropathies (HSN)

Of the six probands with hereditary motor neuropathy (HMN), a genetic diagnosis was determined in only one. A young male proband with difficulty walking since 2 years of age, prominent foot deformities, and a younger sibling who was similarly affected was found by WES to have a known pathogenic heterozygous frameshift *VWA1* variant in exon 1 and a novel likely pathogenic heterozygous frameshift variant in exon 3 (NM_022834.5(VWA1):c.662dup(p.Glu222GlyfsTer65)) [as previously reported (20)]. Nerve conduction studies showed a length-dependent motor axonal polyneuropathy.

In total, three probands with adult-onset chronic small fibre neuropathy, presenting with painful length-dependent neuropathy, skin ulceration, and erythromelalgia, were categorized under the hereditary sensory neuropathy (HSN) phenotype. However, no molecular diagnoses were determined for these probands by WES (*n* = 3).

### 3.3 Complex phenotypes

In total, nine probands (13%) with slowly progressive neuropathies as the main clinical feature, in addition to at least two other syndromic features, were categorized as having “complex phenotypes.” In addition, one individual was clinically classified as having symptomatic sensorimotor neuropathy with hyperreflexia, but electrophysiological studies only showed evidence of chronic motor neurogenic changes on electromyography (see below). A total of two probands of African ancestry, with symptom onset in late adolescence or early adulthood, were diagnosed with autosomal recessive spinocerebellar ataxia with axonal neuropathy and were found by WES to harbor the same *TDP1* variant NM_018319.4(TDP1):c.1478A > G (p.His493Arg). These two individuals were originally from Malawi and Zambia, respectively, but neither reported a known history of consanguinity in their families.

A teenage proband and her sibling, of South Asian ancestry and with parental consanguinity, had signs and symptoms since infancy, including developmental cataracts, glaucoma, short stature, hearing impairment, intellectual disability, seizures, migraines, ataxia, ichthyosis, and sensory axonal neuropathy. They were found to have a homozygous splice donor *PEX11B* variant, which was classified as a VUS. The candidate variant remained classified as a variant of uncertain significance, despite the compelling phenotypic fit consistent with a diagnosis of autosomal recessive peroxisome biogenesis disorder 14B. The highest allele frequency in gnomAD v4.0 was <1 × 10^−4^ among South Asian populations.

Another female proband with childhood-onset gait imbalance, cognitive dysfunction, and frequent pneumonia complicated by bronchiectasis was diagnosed with autosomal recessive ataxia–telangiectasia and was found by WES to harbor a homozygous likely pathogenic missense *ATM* variant. No telangiectasia were detected on examination, but the alpha-fetoprotein level was elevated. A middle-aged proband with symptom onset in late childhood and a sibling with similar symptoms presented with slowly progressive spastic ataxia, tremor, optic atrophy, and premature ovarian insufficiency/gonadal failure. WES identified a homozygous likely pathogenic missense variant in *SERAC1*, consistent with a diagnosis of SERAC1-related neurological disorder presenting as juvenile-onset complicated hereditary spastic paraplegia. The proband did not have some of the other features associated with 3-*me*thyl*g*lutaconic aciduria with *d*eafness-dystonia, (*h*epatopathy), *e*ncephalopathy, and *L*eigh-like syndrome (MEGD(H)EL), such as hearing impairment and liver dysfunction.

An individual of European ancestry with no available family history (both parents and a sibling died of neoplasms in their 40s) was aged 61 years at assessment. He had developed paraesthesia in his feet at age 55, followed by progressively increasing difficulty walking and a need for assistance when climbing stairs. He also complained of instability while showering. Examination showed hyperreflexia, more pronounced in the legs than the arms, without Babinski extensor responses. Although increased tone was not a feature in the legs, the mild proximal leg weakness had an upper motor neuron distribution (hip flexors and abductors weaker than hip extensors and adductors). In addition, muscle wasting was observed in the extensor hallucis brevis muscle, with weakness in the toes. Sensory impairment was present for pin, temperature, vibration and proprioception modalities in a distal stocking distribution. Although electrophysiological studies showed normal distal motor conduction (marginally prolonged tibial nerve F-waves) and normal sural sensory nerve action potentials, electromyography demonstrated chronic neurogenic changes in the distal leg muscles. The participant was found to have a heterozygous *WASHC5* p.Ile831Phe missense VUS, which has previously been described in one individual with SPG8. The patient had a similar, albeit milder, motor phenotype to those previously described (21) but with more prominent sensory changes, which were likely due to pathology proximal to the dorsal root ganglia.

Only one of the four individuals with late adult-onset sensory ataxic neuropathy (SAN) who underwent *RFC1* testing was found to have pathogenic repeat expansions consistent with a diagnosis of cerebellar ataxia with neuropathy and vestibular areflexia syndrome (CANVAS). The one proband with bi-allelic RFC1 AAGGG expansions and European ancestry, also had two other affected family members with bi-alleleic RFC1 AAGGG expansions confirming pseudodominant inheirtance (Figure 2). Features common to the proband (II:4), affected sibling (II:2) and affected nephew (III:3), were length-dependent loss of sensation, ataxia, chronic cough, and pes cavus. RP-PCR screening for *RFC1* expansions showed no amplifiable PCR product and a positive saw-tooth pattern for AAGGG consistent with biallelic expansions in the three affected family members (II:2, II:4, and III:3). In contrast, the unaffected family member tested (II:3) had an amplifiable PCR product and a negative AAGGG RP-PCR result, consistent with the absence of an AAGGG expansion. The affected family members were subjected to Southern blot analysis, which confirmed the presence of biallelic expansions within the pathogenic range.

**Figure 2:**
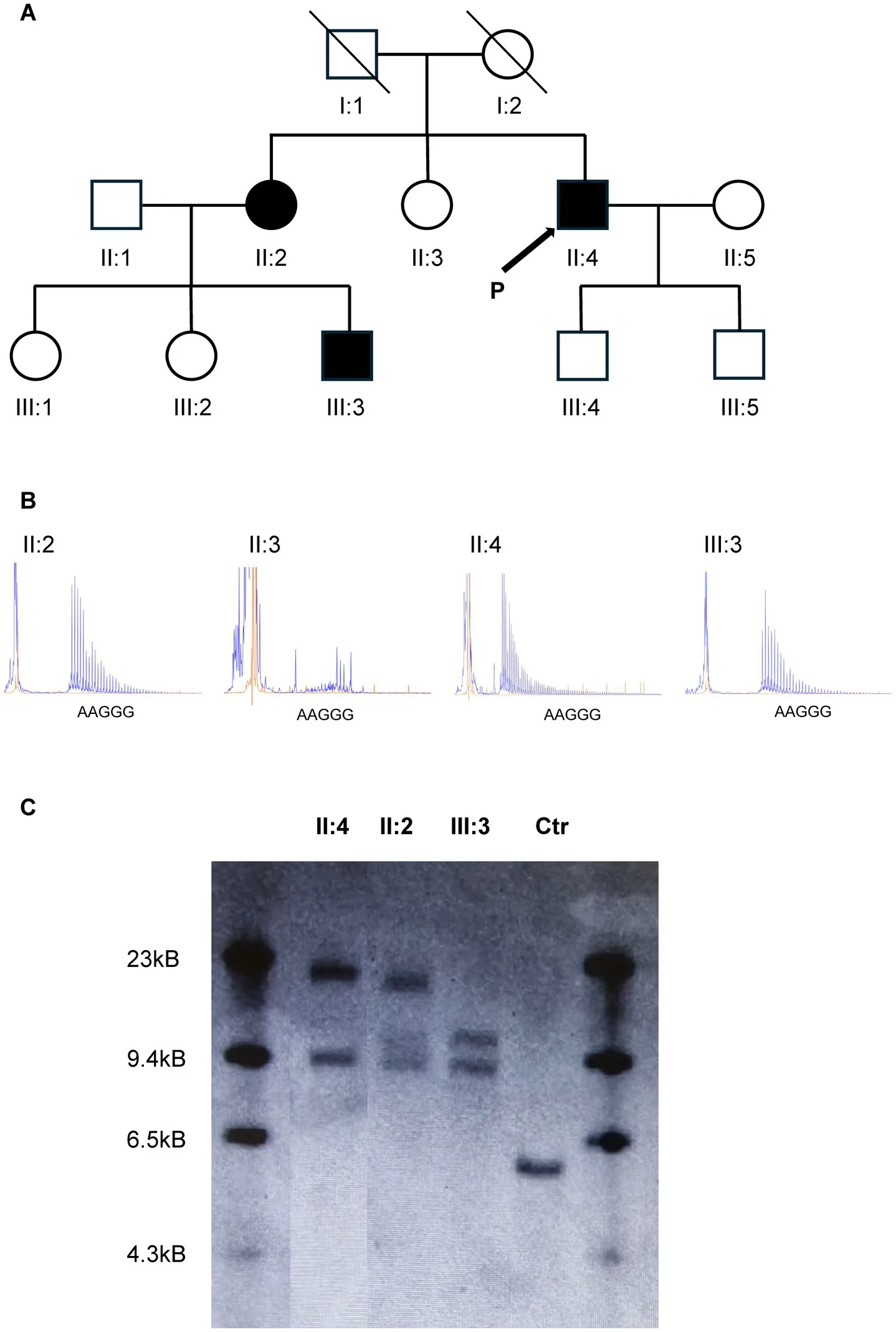
Pseudodominant inheritance pattern in a family of European ancestry with *RFC1* expansions. R*FC1* repeat expansion testing in a family with three individuals affected with peripheral neuropathy. **(A)** P indicates the index patient. **(B)** Repeat-primed PCR for the pathogenic AAGGG configuration in the affected family members (II:2, II:4, and III:3) and the unaffected sibling of the proband (II:3). **(C)** Southern blotting of genomic DNA from the affected family members and an unaffected unrelated control. Patients showed two discrete bands within the expanded range above 6.5 kB.

Overall, the molecular diagnosis rate was similar between the two cohorts (54–56%), despite no CMT1 cases in Zambia (Table 2). In SA, the CMT1 group comprised 71% of participants with CMT1A (*PMP22*), and all reported European ancestry, apart from one participant who reported admixed ancestry. CMT2 was proportionately more frequent in Zambia than in SA (Table 2), but both groups achieved similar molecular diagnostic rates (57% in SA and 55% in ZA). CMT2A (*MFN2*) was proportionately more frequent in Zambia (4/6, all African ancestry) compared to South Africa (3/12, all admixed ancestry), but the group sizes were small, and ancestry reflected the largest subpopulations from that respective center. The complex neuropathy group achieved a diagnosis rate of 67%, compared to 25% for the SAN group. Among the 68 participants in the overall cohort with a genetic diagnosis, the genes harboring CNVs or SNVs in more than one proband were *MFN2* (*n* = 7, 10%), *PMP22* (*n* = 6, 9%), *MPV17* (*n* = 4, 6%), and *TDP1* (*n* = 2, 3%) (Figure 1B). Most of the variants were classified as pathogenic or likely pathogenic (Figure 1C).

## 4 Discussion

Here, we describe the mutational profile of patients with CMT and related hereditary neuropathies from two centers in Southern Africa. Although the cohort is modest in size compared to elsewhere, this study expands the limited data on CMT from sub-Saharan Africa. The most important finding was that axonal neuropathies, or CMT2, were twice as common in these two cohorts of people living with CMT and other hereditary neuropathies in Southern Africa (47%) compared to large cohorts from the UK (19%) and Brazil (22%) (3, 22), using the same electrophysiological classifications. In contrast, demyelinating CMT1 was uncommon (10–14% depending on a conduction velocity cut-off of ≤25 m/s or ≤35 m/s, respectively) and limited to participants with self-reported European or admixed European ancestry in the SA sample; no CMT1 cases were identified among Zambian participants. The proportional distribution of the remaining CMT subtypes viz. CMTi, HMN, HSN and SAN were similar to a large cohort reported from the UK using the same electrophysiological categorization (3). The most frequent cause for axonal CMT in this Southern African cohort was MFN2. MPV17 compound heterozygous variants resulted in a CMT2 or CMTi electrophysiological phenotype (Supplementary Table 3).

Approximately half of this Southern African cohort received a genetic diagnosis, which is lower than the rates reported in larger cohorts from the UK and Brazil, where 60–77% of CMT cases overall received a genetic diagnosis (3, 4). This is likely driven by the high rate of solving CMT1A (*PMP22* CNV) cases. A large multisite North American, European, and Australian cohort reported CMT1A in 62% of cases (1), compared to 81% in the UK (3) and 59% in Brazil (22). None of the probands in this cohort of self-reported African ancestry had CMT1A (*PMP22* CNV) or demyelinating neuropathy on electrophysiological assessment. However, two pediatric patients of self-reported African ancestry with *PMP22*-associated CMT1 were recently reported from South Africa (11). Overall, the most frequent genetic diagnosis among participants of African ancestry was associated with *MFN2* mutations (10%). This was substantially more frequent in the Southern African CMT2 subgroup (22%) than in the reported CMT2 cohorts of European, Brazilian, and Chinese ancestry (4–12%) (1–3, 5, 6, 22, 23).

Compound heterozygous *MPV17* variants (p.Gln36Ter and p.Arg125Trp) were found in four probands from South Africa, but in none from Zambia. The phenotypes of these participants with the same compound heterozygous biallelic *MPV17* variants, confirmed in *trans* in a representative case, were consistent with the CMT2EE clinical picture previously reported in MPV17-associated neuropathy, although previously reported cases harbored homozygous pathogenic variants (Supplementary Table 2). Interestingly, one of the compound heterozygous variants in the SA MPV17-associated neuropathy, p.Gln36Ter, was reported to result in a fatal neurohepatopathy in South African neonates when inherited in a homozygous configuration (24). The fatal neurohepatopathy has also been reported in European individuals with homozygous p.Gln36Pro or p.Arg50Gln (25). Other *MPV17*-associated CMT2EE case reports had similar ages at symptom onset and electrophysiological classifications (either CMT2 or CMTi neuropathy) to those observed in our cohort. These cases harbored the following homozygous *MPV17* variants: p.Arg41Glu [two Korean probands (26)], p.Arg41Gln [two Bosnian siblings (25)], p.Pro98Leu [one Pakistani proband (27)], and homozygous splice variants [c.376-9 T > G; three Iraqi siblings (25)] (see Supplementary Table 2). The *MPV17*-associated CMT2EE case with the homozygous p.Pro98Leu variant had T2/FLAIR hyperintense white matter changes in the centrum semiovale on brain MRI, the clinical relevance of which was unknown when the patient was imaged as a young adult. Functional studies of *MPV17* p.R41Q knockdown showed reduced cell proliferation in neuronal cell lines and impaired mitochondrial function (26). The isiXhosa speaking South African *MPV17* neuropathy cases, in addition to the p.Gln36Ter, all showed co-inheritance of p.Arg125Trp (confirmed in trans in one proband). MPV17 p.Arg125Trp is rare (observed at minor allele frequency (MAF) of 0.0000483, gnomAD v4.1), and currently curated as of unknown significance, but 23-fold more frequent amongst Africans and African-Americans than European population controls.^3^ The MAF of p.Gln36Ter among the South African Black population (specifically in the isiXhosa-speaking communities of the southeastern regions) was 0.007 (24), which is 35-fold higher than the gnomAD v4.1 MAF of 0.0002 reported in African-American populations. We postulate that the high prevalence of the African *MPV17* p.Gln36Ter variant in certain areas of South Africa, together with the more widely distributed African *MPV17* p.Arg125Trp variant, may have increased the likelihood of co-inheritance in the region (South Africa), manifesting as CMT2EE. Although we did not observe these two variants co-segregate among in-house controls, we were unable to perform segregation studies in the affected families. Additional cases in the future will provide supportive evidence for a more definitive classification.

The remaining probands with CMT2 comprised a heterogeneous group, with individual probands harboring variants in different genes, highlighting the need for more comprehensive genetic testing options in our setting. Although *PMP22* copy number variants and pathogenic variants in *GJB1* and *MPZ* accounted for 79% of CMT diagnoses in a multicenter European-ancestry CMT cohort (1) and 64% in a Brazilian sample (4), these variants accounted for only 13% of diagnoses in the South African sample and none in the Zambian sample. Of the South African participants with these variants, six of seven had European ancestry. The proportions of HMN, HSN, and SAN were similar to those reported in larger cohorts (2, 3). One of our families of European ancestry, with a pseudodominant inheritance pattern of SAN and a history of chronic cough, was positive for an *RFC1* expansion. Similar pseudodominant pedigrees have recently been described in Australia (28).

Our complex neuropathy group was proportionately (13%) three times larger compared to the complex neuropathies from a much larger cohort in the UK (*n*>1500; 4%)(3), and may represent an ascertainment bias of more severe phenotypes from our region, recruited from two tertiary referral centers. Although two unrelated probands with *TDP1*-associated autosomal recessive spinocerebellar ataxia with axonal neuropathy were identified among Southern African participants with CMT, this gene did not feature in the large UK cohort (3). Another notable difference between the Southern African cohort and the Global North and Latin American cohorts was the low frequency of *GJB1*, the second most common genetic cause of CMT (6–13% overall), which was detected in only a single European-ancestry CMTX case in this sample (1, 3).

We acknowledge several limitations. Sampling was opportunistic and complicated by limited access to healthcare facilities during the COVID-19 pandemic, which disproportionately affected patients from lower socioeconomic areas. It is likely for this reason that the ancestry distribution in the diverse SA sample from the Western Cape region was skewed toward participants of European ancestry (44%), compared to the approximately 25% expected for this subpopulation in this region. We also acknowledge the potential limitation of relying on self-reported ancestry in the absence of genomic ancestry analyses. Both recruiting centers were situated within tertiary referral centers with electrophysiology laboratories, which may have resulted in more severe cases being referred for evaluation and enrichment of rare phenotypes. To this end, complex neuropathies, although a small subgroup, represented a larger proportion of this selected referral cohort compared to published tertiary cohorts from the Northern Hemisphere (12% vs. 3.5%, respectively) (3), which may reflect ascertainment differences. As expected in understudied populations such as ours, we identified a number of VUS candidates. Although we cannot conclude that these VUS candidates are definitely disease-associated or pathogenic, and experimental functional validation is absent, it is important to catalogue the evidence to advance the field while awaiting the identification of additional cases (see Supplementary Table 4 for the ACMG codes of the VUS). In a resource-constrained setting, functional validation of rare and novel variants is not available. Furthermore, socioeconomic factors and lower life expectancy impact the ability to perform genotype segregation analyses.

Although genetic testing strategies differed between participants based on phenotype, the diagnostic rate achieved, using mainly short-read NGS, was similar to that reported in a recent large UK cohort (3). Despite the relatively small sample size, this study contributes to the limited genetic data on CMT in Africa and may guide future diagnostic testing strategies.

## 5 Conclusion

The mutational spectrum in this phenotypically heterogeneous cohort of Southern African probands with hereditary neuropathies was diverse and different from that reported in the Global North. The main findings were that demyelinating CMT1 was rare in CMT participants of African ancestry and that diagnostic testing should be more comprehensive than *PMP22* genetic testing. Although CMT2 represented the larger subgroup, the genetic diagnostic rate of approximately 50% was similar to that reported globally for CMT2 individuals. *MFN2*- and *MPV17*-related neuropathies were the most frequent causes of CMT among CMT participants of African ancestry.

## Data Availability

The datasets presented in this study can be found in the article/supplementary material. The names of the repository/repositories and accession number(s) can be found in the Supplementary material.

## Footnotes

1
https://www.ilifu.ac.za

2
https://franklin.genoox.com

3
https://www.ncbi.nlm.nih.gov/snp/rs112170670#frequency_tab
