# Supplementary material for: A genetic profile of hereditary neuropathies in two Southern African cohorts
Source: Front Neurol. 2026 Oct 7;17:1918540. doi: 10.3389/fneur.2026.1918540 (PMC13688866; doi:10.3389/fneur.2026.1918540)
Supplement: Supplementary file 1 [file Table_1.docx]

**Supplementary table 1: Demographic and genotypic details of all participants.**

| **Case No.** | **SA/ ZM** | **Sex** | **AAO years** | **Ancestry** | **Positive Family history** | **Test** | **Gene** | **Variant (zygosity)** | **Variant type** | **ACMG** | **ClinVar accession number** |
| --- | --- | --- | --- | --- | --- | --- | --- | --- | --- | --- | --- |
| **CMT1 (n=7)** | | | | | | | | | | | |
| SGS_0008* | SA | F | 8 | ADM | yes | MLPA | *PMP22* | 1.5Mb *PMP22* dup (het) | CNV | P | NA |
| SGS_0055* | SA | M | 9 | EUR | yes | MLPA | *PMP22* | 1.5Mb *PMP22* dup (het) | CNV | P | NA |
| SGS_0075 | SA | M | 15 | EUR | no | MLPA | *PMP22* | 1.5Mb *PMP22* dup (het) | CNV | P | NA |
| SGS _0090 | SA | M | 7 | EUR | yes | MLPA | *PMP22* | 1.5Mb *PMP22* dup (het) | CNV | P | NA |
| SGS_0119 | SA | F | 12 | EUR | yes | MLPA | *PMP22* | 1.5Mb *PMP22* dup (het) | CNV | P | NA |
| SGS_0087 | SA | F | 17 | EUR | yes | srNGS^#^ |  |  |  |  |  |
| SGS_0172 | SA | M | 40 | EUR | no | srNGS^#^ |  |  |  |  |  |
| **CMT2 (n=32)** | | | | | | | | | | | |
| NRG_1 | SA | F | 5 | EUR | yes | lrWGS | *SMN1* | Deletion (het); p.Ile116Thr (het) | CNV, missense | LP | SCV007550383.1 |
| NRG_48* | SA | F | 20 | ADM | yes | srNGS | *MFN2* | p.Thr206Ala (het) | missense | LP | SCV003930375.4 |
| NRG_27* | SA | M | 25 | ADM | no | srNGS | *MFN2* | p.Arg259Cys (het) | missense | P | SCV003930377.4 |
| NRG_29* | SA | F | 10 | ADM | yes | srNGS | *MFN2* | p.Arg280His (het) | missense | P | SCV003930380.5 |
| 391-39 | ZM | F | 5 | AFR | yes | srNGS | *MFN2* | p.Arg94Trp (het) | missense | P | VCV000002276.51 |
| 391-86 | ZM | F | 2 | AFR | no | srNGS | *MFN2* | p.His361Gln (het) | missense | LP | SCV004171119.4 |
| 391-151 | ZM | M | 5 | AFR | yes | srNGS | *MFN2* | p.Arg364Trp (het) | missense | P | SCV005849062.2 |
| 391-187 | ZM | F | 3 | AFR | no | srNGS | *MFN2* | p.Arg94Gln (het) | missense | P | SCV006304456.1 |
| NRG_13* | SA | M | 6 | AFR | no | srNGS | *MPV17* | p.Gln36Ter (het), p.Arg125Trp (het) | stop gain, missense | VUS | SCV003930386.5,SCV003930388.5 |
| SGS_0066* | SA | F | 20 | AFR | no | srNGS | *MPV17* | p.Gln36Ter (het), p.Arg125Trp (het) | stop gain, missense | VUS | SCV003930386.5, SCV003930388.5 |
| SGS_0049* | SA | F | 10 | ADM | yes | srNGS | *GAN* | **p.Gln94Ter (het), p.Pro315Leu (het)** | stop gain | VUS | SCV003930344.5, SCV003930345.4 |
| SGS_0057* | SA | F | 17 | AFR | no | srNGS | *ATP1A1* | p.Ile592Thr (het) | missense | LP | SCV003852622.4 |
| SGS_0060* | SA | F | 40 | ASI | no | srNGS | *RFT1* | p.Gly494Ser (hom) | missense | VUS | SCV003930391.4 |
| SGS_0061* | SA | F | 28 | EUR | yes | srNGS | *ADPRS* | p.Val335Gly (hom) | missense | P | SCV003930343.6 |
| SGS_0148 | SA | M | 9 | AFR | no | srNGS | *HSPB1* | p.Thr180Ile (het) | missense | LP | SCV005921963.1 |
| SGS_0215 | SA | F | 22 | ADM | yes | MLPA | *PMP22* | 1.5Mb *PMP22* del (het) | CNV | P | NA |
| 391-156 | ZM | F | 3 | AFR | no | srNGS | *DYNC1H1* | p.Arg251Cys (het) | missense | LP | SCV005324728.3 |
| NRG_54 | SA | F | 13 | AFR | yes | srNGS |  |  |  |  |  |
| SGS_0018 | SA | F | 16 | AFR | no | srNGS |  |  |  |  |  |
| SGS_0025 | SA | M | 7 | EUR | yes | srNGS |  |  |  |  |  |
| SGS_0027 | SA | M | 16 | EUR | no | srNGS |  |  |  |  |  |
| SGS_0099 | SA | F | 9 | EUR | no | srNGS |  |  |  |  |  |
| SGS_0128 | SA | M | 54 | EUR | no | srNGS |  |  |  |  |  |
| SGS_0132 | SA | F | 26 | AFR | no | srNGS |  |  |  |  |  |
| SGS_0178 | SA | F | 27 | ADM | no | srNGS |  |  |  |  |  |
| SGS_0184 | SA | M | 10 | ADM | no | srNGS |  |  |  |  |  |
| 391-88 | ZM | M | 2 | AFR | yes | srNGS |  |  |  |  |  |
| 391-95 | ZM | M | 11 | AFR | yes | srNGS |  |  |  |  |  |
| 391-129 | ZM | F | 6 | AFR | no | srNGS |  |  |  |  |  |
| 391-135 | ZM | M | 3 | AFR | no | srNGS |  |  |  |  |  |
| 391-204 | ZM | M | 2 | AFR | no | srNGS |  |  |  |  |  |
| 391-93** | ZM | M | 4 | AFR | no | srNGS | *NDRG1* | **NM_006096.4:c.807+1G>T (het),** NM_006096.4:c.755+1G>A (het) | splice donor variants | VUS | SCV005849059.2, SCV005849070.2 |
| **CMTi (n=7)** | | | | | | | | | | | |
| SGS_0031 | SA | M | 18 | AFR | yes | srNGS^#^ | *MPV17* | p.Gln36Ter (het), p.Arg125Trp (het) | stop gain, missense | VUS | SCV003930386.5, SCV003930388.5 |
| SGS_0050* | SA | F | 13 | AFR | no | lrWGS^##^ | *MPV17* | p.Gln36Ter (het), p.Arg125Trp (het) | stop gain, missense | VUS | SCV003930386.5, SCV003930388.5 |
| SGS_0048* | SA | M | 50 | EUR | yes | srNGS^#^ | *MPZ* | **p.Glu71Gly (het)** | missense | VUS | SCV003852624.4 |
| SGS_0067* | SA | M | 14 | ADM | no | srNGS^#^ | *MORC2* | p.Arg252Trp (het) | missense | P | SCV003930346.4 |
| SGS_0110* | SA | F | 11 | EUR | yes | srNGS^#^ | *GJB1* | p.Arg22Ter (het) | stop gain | P | SCV003930347.4 |
| 391-185 | ZM | M | 49 | ASI | yes | SS^§^ | *MME* | p.Gln724Ter (hom) | stop gain | LP | VCV004856389.1 |
| SGS_0160 | SA | M | 13 | AFR | no | lrWGS^#^ |  |  |  |  |  |
| **HMN (n=6)** | | | | | | | | | | | |
| 391-144 | ZM | M | 2 | AFR | yes | srNGS | *VWA1* | p.Gly25ArgfsTer74 (het); p.Glu222GlyfsTer65 (het) | frameshift, frameshift | P, LP | SCV004171120.6, SCV004171121.5 |
| NRG_63 | SA | M | 0 | ADM | yes | srNGS |  |  |  |  |  |
| NRG_19 | SA | M | 19 | ADM | no | srNGS |  |  |  |  |  |
| 391-23 | ZM | M | 14 | AFR | no | srNGS |  |  |  |  |  |
| 391-138 | ZM | M | 1 | AFR | no | srNGS |  |  |  |  |  |
| SGS_0028 | SA | M | 32 | EUR | no | srNGS |  |  |  |  |  |
| **HSN (n=3)** | | | | | | | | | | | |
| SGS_0064 | SA | M | 54 | EUR | no | srNGS |  |  |  |  |  |
| SGS_0151 | SA | F | 20 | AFR | no | srNGS |  |  |  |  |  |
| SGS_0208 | SA | M | 33 | ADM | yes | srNGS |  |  |  |  |  |
| **SAN (n=4)** | | | | | | | | | | | |
| SGS_0043 | SA | M | 60 | EUR | yes | RFC1 assay | *RFC1* | *RFC1* AAGGG repeat expansion (hom) | CNV | P |  |
| SGS_0164 | SA | M | 55 | EUR | no | RFC1 assay |  |  |  |  |  |
| SGS_0170 | SA | M | 75 | EUR | no | RFC1 assay |  |  |  |  |  |
| SGS_0209 | SA | M | 57 | EUR | no | RFC1 assay |  |  |  |  |  |
| **Complex (n=9)** | | | | | | | | | | | |
| SGS_0021* | SA | F | 5 | ADM | yes | srNGS | *ATM* | p.Thr1743IIe (hom) | missense | P | SCV003930348.4 |
| SGS_0163 | SA | F | 12 | ADM | yes | srNGS | *SERAC1* | **p.Pro533Leu (hom)** | missense | LP | SCV004704616.4 |
| SGS_0179 | SA | M | 15 | AFR | yes | srNGS | *TDP1* | p.His493Arg (hom) | missense | P | SCV005849061.3 |
| 391-121 | ZM | M | 25 | AFR | yes | srNGS | *TDP1* | p.His493Arg (hom) | missense | P | SCV005849061.3 |
| SGS_0190 | SA | F | 0 | ASI | yes | srNGS | *PEX11B* | NM_003846.3(PEX11B):c.172+1G>A (hom) | splice donor | VUS | SCV005921962.1 |
| NRG_28 | SA | M |  | ADM | yes | srNGS |  |  |  |  |  |
| SGS_0019 | SA | M | 2 | EUR | yes | srNGS |  |  |  |  |  |
| SGS_0120 | SA | M | 38 | EUR | yes | srNGS |  |  |  |  |  |
| SGS_0131 | SA | M | 55 | EUR | no | srNGS | *WASHC5* | p.Ile831Phe (het) | missense | VUS | VCV003760738.3 |

Legend. Charcot-Marie-Tooth (CMT) and related neuropathies identified in cohorts from South Africa (SA) and Zambia (ZM). Male (M); female (F). Age at onset (AAO). “Ancestry” refers to self-reported race categories: African (AFR), European (EUR), admixed (ADM) and Asian (ASI) ancestry. Charcot-Marie-Tooth (CMT); hereditary sensory neuropathy (HSN); hereditary motor neuropathy (HMN); sensory ataxic neuropathy (SAN); hereditary neuropathy with liability to pressure palsies (HNPP); “complex” refers to cases with at least one non-neuropathy symptom or sign. Het refers to heterozygous; dup to duplication; del to deletion, dom to dominant. Test refers to the gene assay that allowed a genetic diagnosis; Multiplex ligation-dependent probe amplification (MLPA), srNGS refers to short read next generation sequencing that includes either whole exome and/or whole genome sequencing (WGS). lrWGS to long read WGS. SS refers to Sanger sequencing; CNV to copy number variation. American College of Medical Genetics and Genomics (ACMG) classification: Pathogenic (P), likely pathogenic (LP) and variant of uncertain significance (VUS). The VUS were considered to be strongly phenotype-matched with biological plausibility and retained as candidate explanatory variants until reclassification may follow with additional cases, functional data, or segregation studies. Variants that are novel for the phenotype are indicated in bold text although some *variants have been reported by the same group (10). **this participant is presumed to have severe axonal neuropathy (see text). ^#^*PMP22* MLPA negative. ^##^lrWGS was performed in one individual with *MPV17* to confirm a compound heterozygous trans configuration. ^§^sibling had undergone srNGS previously. Variants in genes routinely assessed through established diagnostic testing pathways were excluded from ClinVar submission and indicated as NA or not applicable. The blank cells in the gene column indicates a genetically unsolved case.
