# Supplementary material for: A genetic profile of hereditary neuropathies in two Southern African cohorts
Source: Front Neurol. 2026 Oct 7;17:1918540. doi: 10.3389/fneur.2026.1918540 (PMC13688866; doi:10.3389/fneur.2026.1918540)
Supplement: Supplementary file 2 [file Table_2.docx]

**Supplementary table 2: Phenotypes reported as CMT2EE with homozygous *MPV17* pathogenic variants and compared to South Africans cases with compound heterozygous *MPV17* variants.**

| reference | (26) | | (27) | (25) | | SGS_0050 | SGS_0066 | SGS_0031 | NRG_13 |
| --- | --- | --- | --- | --- | --- | --- | --- | --- | --- |
| Patient/family No. | FC26 | FC355 |  | F1, n=2 | F2, n=3 |  |  |  |  |
| AAO (years) | 9 | 13 | <1 | 8-10 | 15-23 | 13 | 20 | 18 | 6 |
| Consanguinity (ancestry) | No (Korean) | | No (Pakistan) | No (Austria) | No (Iraq) | No (South African Black-Xhosa) | | | |
| Laryngeal palsy with stridor | NR | NR | NR | NR | NR | - | - | - | + |
| Sensorineural hearing loss | - | - | + | + (n=1) | - | - | - | + | - |
| Hand tremor | NR | NR | + | NR | NR | + | - | - | - |
| Distal upper limb weakness | + | + | + | + | + | + | + | + | + |
| Clawed hands | + | + | - | - | - | - | - | + | - |
| Numbness of hands and legs | - | - | + | + | + | + | - | - | + |
| Areflexia | + | + | + | + | + | + | + | + | + |
| Distal limb muscle atrophy | + | + | + | + | + | + | + | + | + |
| Foot drop | + | + | + | - | + | + | + | + | + |
| Pes cavus | - | - | - | + | - | - | - | + | - |
| Clawed toe | + | + | + | + | + | - | - | + | - |
| Scoliosis | + | NR | - | NR | NR | - | - | + | - |
| Walking difficulties | + | + | + | + | + | + | + | + | + |
| other | - | - | - | - | NIV n=1^#^ | - | - | - | - |
| Axonal sensorimotor neuropathy | slowing  CMTi | + | + | + | + | slowing  CMTi | + | slowing/  CMTi | + |
| Sequencing approach | WES | | WES | WES | WES | Short read WGS, 1 long read WGS | | | |
| *MPV17* variant (OMIM: 137960) | p.R41Q (hom) | | p.P98L (hom) | p.R41G (hom) | c.376-9T>G  (hom) | p.G36* (het); p.R125W (het) | | | |
| Segregation | yes | yes | UK | UK | Yes | UK | UK | UK | UK |

Legend: NR refers to not reported, UK as unknown. WES refers to whole exome sequencing, WGS to whole genome sequencing. Hom refers to homozygous and Het to heterozygous. ^#^non-invasive nocturnal ventilation for respiratory muscle weakness in one individual at age 43.
