# Supplementary material for: A genetic profile of hereditary neuropathies in two Southern African cohorts
Source: Front Neurol. 2026 Oct 7;17:1918540. doi: 10.3389/fneur.2026.1918540 (PMC13688866; doi:10.3389/fneur.2026.1918540)
Supplement: Supplementary file 3 [file Table_3.docx]

**Supplementary table 3. Electrophysiological features of participants with compound heterozygous candidate VUS in *MPV17***

| Patient | NRG_13 | SGS_0050 | | SGS_0031 | | SGS_0066 | Normal values |
| --- | --- | --- | --- | --- | --- | --- | --- |
| Age at exam (years) | 16 | 26 | | 32 | | 38 |  |
| Side | Left | Right | Left | Right | Left | Left |  |
| Median nerve  DML (ms)  CMAP (mV)  MNCV (m/s) | 3.7  9.0  50.4 | **4.8**  **2.5**  **43.7** | **6.6**  **0.6**  48.7 | **8.5**  **0.6**  **34.5** | **5.3**  **2.4**  **37.0** | 3.9  21.4  56.3 | <4.5  >4  >48 |
| Ulnar nerve  DML (ms)  CMAP (mV)  MNCV (m/s) | 3.2  7.1  **49.8** | 3.5  **0.2**  **48.1** | 3.5  **2.4**  57.0 | **A**  **A**  **A** | **5.3**  **0.6**  **46.6** | 3.1  16.5  58.4 | <3.6  >6  >51 |
| Peroneal nerve  DML (ms)  CMAP (mV)  MNCV (m/s) | 5.0  **0.9**  **A** | ND | **A**  **A**  **A** | **A**  **A**  **A** | ND | **A**  **A**  **A** | <5.9  >2  >41 |
| Tibial nerve  DML (ms)  CMAP (mV)  MNCV (m/s) | **A**  **A**  **A** | ND | **A**  **A**  **A** | **A**  **A**  **A** | ND | **A**  **A**  **A** | <6  >4  >41 |
| Median sensory nerve  SNAP (μV)  SNPL (ms) | 83.3  2.2 | **A**  **A** | **10.9**  1.9 | **A**  **A** | ND | 52.5  2.1 | >50  <2.2 |
| Ulnar sensory nerve  SNAP (μV)  SNPL (ms) | 19.4  2.1 | **A**  **A** | **A**  **A** | **A**  **A** | ND | 15.6  2.1 | >15  <2.1 |
| Sural nerve  SNAP (μV)  SNPL (ms) | **A** | ND | **A** | **A** | ND | **A** | >6  <5.4 |

Legend: A, absent response; CMAP, compound muscle action potential; DML, distal motor latency; MNCV, motor nerve conduction velocity; SNAP, sensory nerve action potential; SNPL, sensory nerve peak latency. ND refers to not done. Bold character indicates abnormal values.
