# Supplementary material for: A genetic profile of hereditary neuropathies in two Southern African cohorts
Source: Front Neurol. 2026 Oct 7;17:1918540. doi: 10.3389/fneur.2026.1918540 (PMC13688866; doi:10.3389/fneur.2026.1918540)
Supplement: Supplementary file 4 [file Table_4.docx]

**Supplementary table 4 describing the ACMG/AMP criteria supporting each candidate variant of uncertain significance (VUS).**

| **Case No(s).** | **Gene** |  | **ACMG/AMP evidence criteria supporting VUS classification** |
| --- | --- | --- | --- |
| SGS_049 | *GAN* | p.Gln94Ter (het) | PM2_supporting: this variant is absent from gnomAD v2.1.1 and v3.1.2 (adequate coverage >20X confirmed) and an internal database of 1074 control alleles. PVS1 met: this nonsense (stop gained) variant is found in exon 2 of the *GAN* gene which has a total of 11 exons. It is predicted to result in nonsense mediated decay. Loss of function of at least one *GAN* allele is an established disease mechanism for Giant Axonal Neuropathy (>=35% of pathogenic variants reported in *GAN* are LOF across more than one exon, GAN null mice recapitulate characteristic human GAN features) |
|  | *GAN* | p.Pro315Leu (het) | PM2_supporting: the highest population allele frequency in gnomAD v4.0 is 0.0008910 (0.089%; 57/63974 alleles in European Finnish population) and in gnomAD v3.1.2 is 0.0007556 (0.076%; 1/10588 alleles in European Finnish population) and the variant is absent from an internal database of 1074 alleles. PS4_supporting: variant identified in 2 unrelated probands with consistent phenotype for disorder (PMID 14718689, PMID 17578852). PP3 not met: REVEL score is 0.49. |
| SGS_060 | *RFT1* | p.Gly494Ser (hom) | PM2_supporting: the highest population allele frequency in gnomAD v4.0 is 0.00009799 (0.0098%; 3/30616 alleles in South Asian population) and the variant is absent in gnomAD v3.1.2. is 0.0004798 (0.048%; 1/2084 alleles in Other population) and the variant is absent from an internal database of 1074 control alleles. PP3 met: REVEL score is 0.70. PM3 met: 0.5 points awarded for homozygous observation of variant in proband under assessment. |
| UTH_093 | *NDRG1* | NM_006096.4:c.807+1G>T (het) | PM2_supporting: variant is absent from gnomAD v4.0 (adequate coverage >20X confirmed) and an internal database. PVS1 met: canonical GT-AG splice site variant predicted to cause exon skipping or use of a cryptic splice site which disrupts the reading frame and is predicted to undergo NMD. Exon is present in biologically-relevant transcript(s). PM3 not met: proband under assessment also has heterozygous NM_006096.4(NDRG1):c.755+1G>A variant (ClinVar VCV001068301.6) which is classified as a variant of uncertain significance due to insufficient pathogenic evidence. This variant has not been confirmed in trans with the variant under assessment in the proband- 0 points. |
|  | *NDRG1* | NM_006096.4:c.755+1G>A (het) | PM2_supporting: variant is absent from gnomAD v4.0 (adequate coverage >20X confirmed) and an internal database. PS4 not met: Variant detected in the heterozygous state with no second reportable NDRG1 variant. It was identified in a healthy adult in the context of carrier screening for reproductive purposes (Invitae, ClinVar SCV001577683.3). PVS1_moderate: GT-AG splice site variant which is predicted to cause exon skipping or use of a cryptic splice site. Role of region in protein function is unknown splice site preserves reading frame. LOF variants in this exon are not frequent in the general population and exon is present in biologically-relevant transcript(s). Variant removes <10% of protein. |
| SGS_190 | *PEX11B* | NM_003846.3(PEX11B):c.172+1G>A (hom) | The highest population allele frequency in gnomAD v4.0 is 0.00004463 (4/89620 alleles in the South Asian population) and there are no homozygous observations. PM3_supporting: 0.5 points awarded for homozygous observation of variant in proband under assessment. PVS1 met: GT-AG splice sites, exon skipping or use of a cryptic splice site disrupts reading frame and is predicted to undergo NMD, exon is present in biologically-relevant transcript(s). Loss of function is a known mechanism of disease (PMID:28129423,35624152). |
| NRG13  SGS_066  SGS_031  SGS_050 | *MPV17* | p.Gln36Ter (het) | PM2_supporting: the highest population allele frequency in gnomAD v4.0 is 0.00020 (0.02%; 5/24944 alleles in African/African American population) and the variant is absent from an internal database of 1074 control alleles. PP3_met: Revel score is 0.657. |
|  | *MPV17* | p.Arg125Trp (het) | PM2_supporting: this variant is absent from gnomAD v4.0 (adequate coverage >20X confirmed) while it has a MAF of 0.007 and 0.015 in two independent control samples from South Africa (PMID 29318572, PMID 30782936). PVS1 not assigned as loss of gene function is not an established pathogenic mechanism for MPV17-related CMT2EE; the majority of MPV17 variants associated with CMT2EE are homozygous missense variants while the single reported homozygous non-frameshift deletion, p.Asp126_Tyr136del, removes <10% of the MPV17 protein. |
| SGS_131 | *WASHC5* | p.Ile831Phe (het) | PM2_supporting: The highest population allele frequency in gnomAD v4 is 0.00009601 (0,002%, 6/62496 alleles in the remaining population), no homozygous observations were noted. The variant is absent from AGVD. PP3_Moderate: Revel score is 0.80. PM1 not met: Pathogenic variants cluster in the central spectrin-repeat region of strumpellin (approximately residues 241–791) (PMID:31814071). |
| SGS_048 | *MPZ* | p.Glu71Gly (het) | PM2_supporting: this variant is absent from gnomAD v4.0 (adequate coverage >20X confirmed) and an internal database of 1074 control alleles. BP4 met: REVEL score 0.23. PP1 not met: 2 informative meioses in 1 family. PM1 met: variant occurs in the immunoglobulin (Ig)-like extracellular domain with other pathogenic variants. PM5 not met: MPZ p.Glu71Lys (PMID: 34060176) and p.Glu71Asp (ClinVar SCV000552947.4) variants classified as variants of uncertain significance. |
